# Flexoelectric Suppression of Interfacial Carrier Loss in BaTiO_3_ Thin‐Film Bulk Photovoltaic Systems

**DOI:** 10.1002/advs.76848

**Published:** 2026-07-29

**Authors:** Minwoo Jang, Hyunkyu Lim, Sanghoon Yeom, Jaewhan Oh, Yongsoo Yang, Hyungwoo Lee

**Affiliations:** ^1^ Department of Energy Systems Research Ajou University Suwon Republic of Korea; ^2^ Department of Physics Korea Advanced Institute of Science and Technology (KAIST) Daejeon Republic of Korea; ^3^ Graduate School of Semiconductor Technology School of Electrical Engineering Korea Advanced Institute of Science and Technology (KAIST) Daejeon Republic of Korea; ^4^ Department of Physics Ajou University Suwon Republic of Korea

**Keywords:** BaTiO_3_, bulk photovoltaic, carrier recombination, flexoelectric, interfacial loss, strain gradient

## Abstract

Bulk photovoltaic (BPV) effects in ferroelectric oxides have attracted significant attention for their potential to circumvent the bandgap‐limited open‐circuit voltage of conventional solar cells. However, the practical realization of thin‐film BPV devices remains challenging due to interfacial carrier loss, which severely limits photocurrent. Here, we demonstrate in single‐crystalline BaTiO_3_ thin films that mechanically induced flexoelectric strain gradients effectively suppress interfacial carrier recombination. By combining systematic loading experiments with self‐consistent electrostatic modeling, we show that strain gradients reconfigure interfacial electrostatics, thereby enhancing the local drift field. This flexoelectric modulation substantially increases photocurrent by mitigating interfacial carrier loss, resulting in a ∼35‐fold (3557%) increase in photocurrent. Our modeling framework quantitatively captures the thickness‐dependent carrier extraction and further reveals that structural, electrostatic, and materials‐specific parameters cooperatively govern the extraction efficiency in interface‐limited BPV systems. Therefore, these results provide a physically grounded pathway to interfacial loss engineering in thin‐film BPV systems through flexoelectric modulation.

## Graphical abstract

In single‐crystalline BaTiO_3_ thin films, flexoelectric strain gradients strongly enhance photocurrent by suppressing interfacial carrier loss. Loading experiments reveal a ∼35‐fold photocurrent increase with applied force. Self‐consistent electrostatic modeling shows that strain gradients reshape the interfacial potential landscape, strengthen local drift fields, and govern carrier‐extraction efficiency, establishing flexoelectric modulation as a route to interfacial‐loss engineering in bulk photovoltaic systems.

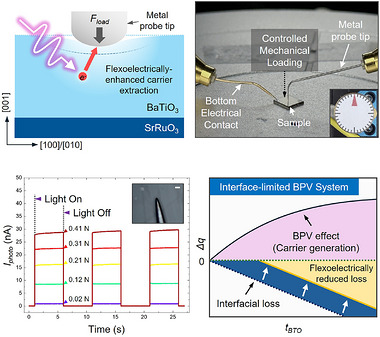

## 1 Introduction

The bulk photovoltaic (BPV) effect enables photocurrent generation in non‐centrosymmetric crystals without a p‐n junction [1, 2]. Owing to its ability to generate photovoltages beyond the bandgap limit of conventional solar cells, the BPV effect has attracted considerable attention [3, 4, 5]. In ferroelectric oxides such as BaTiO_3_ (BTO) and BiFeO_3_, the BPV photocurrent is generally attributed to shift‐current mechanisms arising from second‐order nonlinear optical responses [6, 7]. The intrinsic shift‐current density can be expressed as:

(1) $$J_{\mathrm{shift}}=e\sum_{n,m}W_{nm}R_{nm}$$

where *W*_nm_ is the optical transition rate from band *m* to band *n* and *R*_nm_ is the real‐space shift vector associated with inter‐band transitions. This intrinsic generation term is governed by crystal symmetry and electronic structure [7, 8, 9]. Accordingly, most previous studies have focused on enhancing nonlinear optical generation through polarization engineering, symmetry control, and band‐structure optimization in order to increase the photocurrent [10, 11, 12, 13, 14]. However, this generation‐centric perspective implicitly assumes that photocarrier extraction is sufficiently efficient once carriers are created. In practical thin‐film implementations, photocarriers generated within the film must traverse the bulk and escape through metal/ferroelectric interface before being collected. This interface can act as recombination‐active boundaries, such that the extracted short‐circuit current density (*J*_sc_) is no longer governed solely by intrinsic generation mechanism [15].

The effective carrier lifetime in ferroelectric thin films (τ_eff_) can be written as:

(2) $$\frac{1}{\tau_{\mathrm{eff}}}=\frac{1}{\tau_{\mathrm{bulk}}}+\frac{1}{\tau_{\mathrm{intf}}}$$

where τ_bulk_ and τ_intf_ are the intrinsic bulk lifetime and the interfacial recombination lifetime of the photogenerated electrons, respectively. When τ_intf_ ≪ τ_bulk_, the effective lifetime collapses to τ_eff_ ≈ τ_intf_, and carrier extraction becomes limited by interfacial loss rather than bulk recombination. In this regime, enhancing photogeneration does not guarantee proportional enhancement of the collected photocurrent. More generally, the short‐circuit photocurrent density for a film of thickness *t* can be written as:

(3) $$J_{sc}\;\left(t\right)=\int_{0}^{t}g_{photo}\left(z\right)C\left(z\right)dz\;$$

where *z*, *g*_photo_(*z*), and *C*(*z*) are the depth from the illuminated film surface, the local photogeneration rate, and the collection probability for carriers generated at depth *z*, respectively. When interfacial recombination is strong, the collection probability is largely determined by carrier survival near the interface. The overall photocurrent is then highly sensitive to interfacial carrier extraction efficiency (i.e., *C*(*z*))) [8, 16], even if photogeneration within the bulk is intrinsically strong. Therefore, in thin‐film BPV systems, interfacial loss often becomes the dominant bottleneck that limits the measurable photocurrent, implying that improving interfacial carrier extraction is a key strategy for enhancing BPV performance.

Here, we demonstrate that mechanically induced flexoelectric polarization can substantially reduce interfacial loss in thin‐film BPV systems. Using single‐crystalline BTO thin films as a model system, we show that strain gradients generated by mechanical loading reshape the local electrostatic potential near the metal/BTO interface, leading to suppressed interfacial carrier recombination without affecting intrinsic photogeneration or leakage conduction. When the loading force increases from 0.02 N to 0.41 N, the *J_sc_* increases by more than thirty‐fold (3557%), highlighting the strong sensitivity of photocurrent extraction to interfacial loss. Self‐consistent electrostatic calculations further reveal that strain‐gradient‐induced polarization enhances local electron drift near the interface, thereby reducing recombination probability. These results identify strain‐gradient engineering as a direct and effective route to overcoming interfacial loss in thin‐film BPV systems.

## 2 Controlling Interfacial Loss via Mechanically Induced Strain Gradients

**FIGURE 1:**
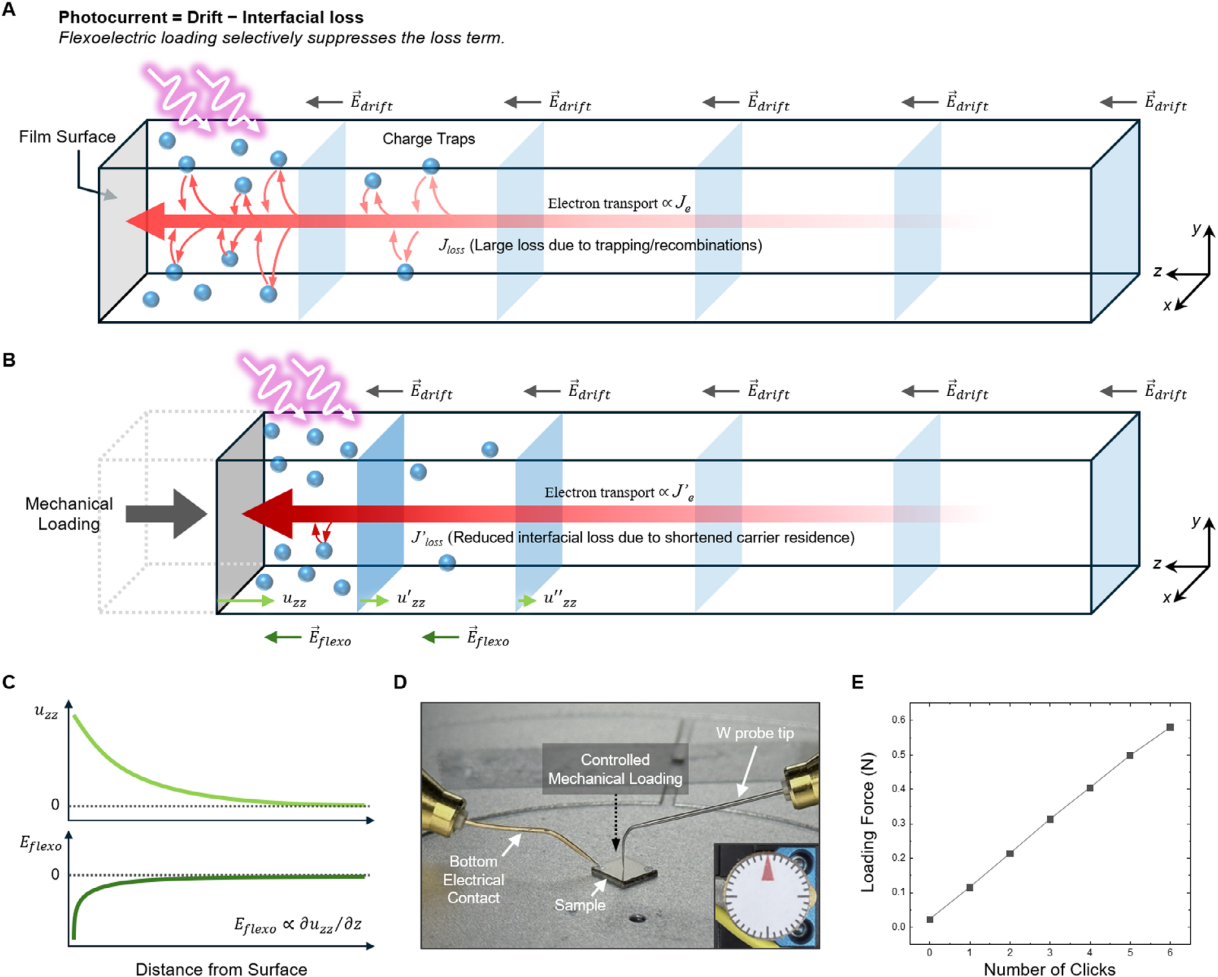
Controlling interfacial loss by mechanically induced strain gradients in thin‐film bulk photovoltaic (BPV) systems. (a) Schematic illustration of carrier transport in an interface‐limited BPV system. In the absence of mechanical loading, photogenerated carriers experience significant interfacial trapping and recombination, leading to large interfacial loss (*J*_loss_) during drift transport. (b) Under mechanical loading, a strain gradient generates a flexoelectric field (*E*_flexo_) near the surface, enhancing the local drift field (*E*_drift_) and shortening the carrier residence time in the recombination‐active region, thereby reducing interfacial loss (*J’*_loss_). (c) Depth dependence of the out‐of‐plane strain (*u_zz_*) generated by localized W‐tip loading and the corresponding *E*_flexo_. (d) Optical image of the experimental setup, showing the W‐probe tip used for controlled mechanical loading and photocurrent measurements. (e) Calibration of the applied loading force as a function of the knob rotation (number of clicks).

Figure 1a schematically illustrates how interfacial loss limits photocurrent extraction in BTO‐based thin‐film BPV systems. In metal/BTO/metal heterostructures, ferroelectric polarization together with the intrinsic band alignment give rise to a finite internal drift field (*E*_drift_), which drives photogenerated electrons toward the collecting metal electrode (Figure S1). However, not all photogenerated electrons can be collected, because some electrons are lost through trapping and recombination during transport. In particular, recombination near the metal/ferroelectric interface plays a dominant role in limiting the photocurrent [17, 18]. In BTO thin films, the surface and interfacial regions inherently contain charged point defects, commonly associated with oxygen vacancies [19, 20]. The shallow defect states introduced by the oxygen vacancies can serve as Shockley‐Read‐Hall recombination centers [21], making trap‐assisted recombination generally active in the vicinity of the metal/BTO interface. Therefore, electrons photogenerated beyond a finite depth contribute only weakly to the effective photocurrent, even if optical absorption remains significant. This provides a natural explanation for why increasing the film thickness does not necessarily result in proportional enhancement of photovoltaic performance.

Based on this physical picture, we propose that interfacial recombination can be substantially mitigated by reshaping the electrostatic potential distribution near the interface, as illustrated in Figure 1b. When mechanical loading is applied to the surface of the BTO thin film, an out‐of‐plane strain gradient is generated, giving rise to a flexoelectric polarization [22, 23, 24, 25, 26]. This strain gradient arises because the mechanical deformation is largest near the W‐tip contact at the BTO surface and decays toward the bottom SRO electrode due to elastic relaxation and substrate clamping. The strain‐gradient‐induced polarization modifies the local electrostatic potential and redistributes the internal electric field near the metal/BTO interface. If the induced field enhances the local electron drift and reduces their residence time in the recombination‐active region, the probability of trap‐assisted recombination can be effectively suppressed.

Under mechanical loading, the out‐of‐plane strain component ε_*zz*_ varies strongly with depth position (*z*) (the upper panel of Figure 1c), inducing a pronounced strain gradient near the surface. Such a strain gradient induces a flexoelectric polarization [27], which can be written as:

(4) $$P_{flexo\_z}=\mu_{eff}\;\frac{\partial \varepsilon_{zz}}{\partial z}$$

where $P_{flexo\_z}$, ε_*zz*_, and µ_*eff*_ are the out‐of‐plane component of flexoelectric polarization, the normal strain along the *z*‐direction, and an effective flexoelectric coefficient representing the dominant tensor contribution under the applied loading configuration, respectively. Given the thin film geometry, we only focus on the out‐of‐plane (i.e., the *z*‐axis) flexoelectric response. The spatial variation of $P_{flexo\_z}$ leads to bound charges (ρ_*b*_ =   − ∇ · *P_flexo_*), which generate an additional internal electric field *E_flexo_* (*z*) near the metal/BTO interface (the lower panel of Figure 1c). This flexoelectrically induced field reshapes the local electrostatic potential landscape and thereby enables modulation of electron recombination dynamics in the interfacial region.

To experimentally investigate this mechanism, we prepared BaTiO_3_/SrRuO_3_/SrTiO_3_ (BTO/SRO/STO) heterostructures. The BTO and SRO thin films were epitaxially grown on TiO_2_‐terminated STO (001) substrates by pulsed laser deposition (PLD) (Figure S2) [19]. The metallic SRO layer served as the bottom electrode, while a commercial tungsten (W) probe tip was used as the top contact, simultaneously applying mechanical loading during photocurrent measurements (Figure 1d). The mechanical loading applied by the W‐probe was carefully controlled to enable quantitative investigation of the flexoelectric response. A custom‐built tuning knob was employed to regulate the probe displacement (inset of Figure 1d), and the corresponding loading force was calibrated as shown in Figure 1e (see Figure S3 for details). We note that irreversible surface damage was observed when the applied force exceeded approximately 0.5 N. Therefore, all measurements in this study were performed under loading forces below this threshold. This controlled loading configuration enables systematic investigation of how strain‐gradient‐induced electrostatic modulation influences photocurrent extraction under optical excitation.

**FIGURE 2:**
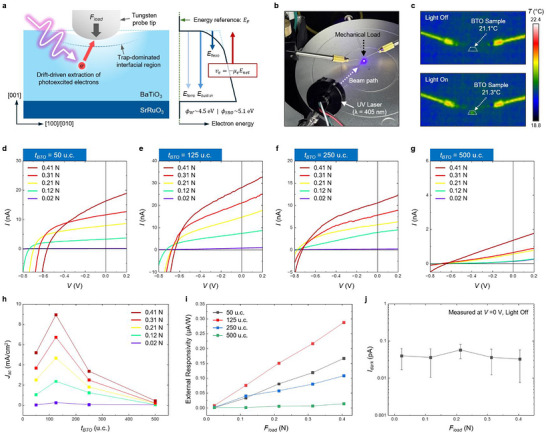
Enhanced photocurrent in BTO thin films through flexoelectric modulation of interfacial electrostatics. (a) Schematic illustration of the experimental configuration. A W‐probe tip applies a controlled mechanical load (*F*_load_) to the BTO surface while serving as the top electrical contact. The strain gradient near the interface generates a flexoelectric field, which accelerates extraction of photogenerated carriers from the trap‐dominated interfacial region. The right panel illustrates the corresponding band diagram modification under flexoelectric modulation. (b) Optical image of the measurement setup under UV illumination. (c) Infrared thermal images of the BTO sample under light‐off and light‐on conditions, confirming negligible photothermal heating during measurements. (d–g) *I*–*V* characteristics under varying mechanical loads for BTO films with thicknesses of 50, 125, 250, and 500 unit‐cells (u.c.), respectively. Increasing *F*_load_ systematically enhances the photocurrent, with the most pronounced enhancement observed at 125 u.c. (h) Short‐circuit current density (*J*_sc_) as a function of film thickness *t*_BTO_ for different *F*_load_, revealing a non‐trivial thickness dependence and the emergence of an optimal thickness under flexoelectric modulation. (i) External responsivity as a function of *F*_load_ for different *t*_BTO_, demonstrating load‐dependent enhancement of carrier extraction efficiency. (j) Dark current measured at *V* = 0 V under different *F*_load_. The absence of *F*_load_‐dependence confirms that the photocurrent enhancement does not originate from geometric enlargement of the contact area.

To directly evaluate the flexoelectric effect on the photocurrent, we illuminated the BTO sample with UV light (*λ* = 405 nm). As discussed above, the intrinsic drift field in BTO drives the photoexcited electrons toward the collecting metal contact (Figure 2a). Considering the polarity of the built‐in field in the W/BTO/SRO heterostructure, the photoexcited electrons are collected by the top W tip (see the simplified energy band diagram in Figure 2a). During this carrier extraction process, all photogenerated electrons must inevitably traverse the trap‐dominated interfacial region. Therefore, photocurrent loss arising from recombination and trapping is determined by how efficiently and rapidly photogenerated electrons pass through this interfacial region. In this context, the flexoelectric field induced at the W/BTO interface is expected to modify the local interfacial drift, thereby regulating the recombination probability. Figure 2b shows the experimental setup inside the shielded probe station. During the measurements, the chamber was kept completely dark, and only the UV laser was modulated, enabling precise evaluation of the intrinsic BPV characteristics of BTO thin films. Before proceeding further, we verified that photothermal effects are negligible in this system. We confirmed that the temperature of BTO samples remains nearly identical with and without UV illumination (Figure 2c), indicating that the measured current originates predominantly from the BPV effect.

Based on this experimental setup, we examine the *I–V* characteristics of BTO thin films under UV illumination and controlled mechanical loading. Figure 2d–g show the *I–V* curves measured for BTO films with thicknesses of 50, 125, 250, and 500 unit‐cells (u.c.). Before the first measurement, the ferroelectric polarization was poled downward to maximize the photocurrent (Figure S4). The measured *I‐*‐*V* curves clearly show that increasing the applied mechanical load systematically enhances the photocurrent. For thinner films (50 and 125 u.c.), the enhancement under mechanical loading is pronounced, indicating that modulation of the interfacial drift field effectively suppresses recombination losses. As the film thickness increases (250 and 500 u.c.), however, the relative enhancement becomes weaker, suggesting that additional transport and recombination processes within the bulk contribute to the overall photocurrent behavior. The observed thickness dependence requires careful interpretation. The relatively weaker enhancement in thicker films mainly arises from the reduced absolute magnitude of the photocurrent. As the film thickness increases, recombination processes within the bulk region become increasingly significant in addition to interfacial recombination. These bulk recombination effects, together with interfacial losses, collectively determine the net photocurrent. Therefore, simply increasing the film thickness does not necessarily result in enhanced photocurrent. Instead, the results suggest the existence of an optimal thickness that maximizes photovoltaic efficiency. In this study, to isolate and clarify the role of interfacial modulation, we focus on the electron dynamics in the 125‐u.c.‐thick BTO film.

Figure 2h,i show the *J_sc_* and external responsivity (*R*) extracted from the *I–V* datasets, respectively. The *J_sc_* values were calculated by normalizing *I*_sc_ to the circular contact area of W‐probe tip (π*a* ^2^ ≈ 3.14 × 10^−6^ cm^2^). It is evident that the enhancement in *J*_sc_ induced by mechanical loading exhibits a non‐trivial thickness dependence in the BTO thin films. Notably, at a thickness of 125 u.c. (*t* ∼50 nm), *J*_sc_ increases by as much as 3557%, indicating substantial suppression of recombination losses. The responsivity *R* exhibits a similar trend, further confirming that mechanical loading significantly improves the BPV performance of BTO thin films. The nearly linear dependence of *R* on loading force (*F*_load_) reveals the strong sensitivity of photocurrent extraction to mechanical modulation. The original *I*_sc_ and the corresponding external quantum efficiency (EQE) as a function of *F*_load_ are provided in Figure S5. Importantly, in contrast to the photocurrent, the intrinsic dark current does not exhibit a proportional dependence on *F*_load_ (Figure 2j). The dark *I*‐*V* characteristics also show that the forward‐bias conduction and the overall diode‐like behavior remain essentially unchanged under different *F*_load_ (Figure S6). These results rule out simple contact‐area enlargement or barrier‐modification effects as the origin of the mechanically enhanced photocurrent. Additionally, to exclude mechanically induced domain reconfiguration as the origin of the enhanced photocurrent, we performed piezoresponse force microscopy (PFM) measurements on the same downward‐poled BTO region before and after W‐tip loading. The nearly unchanged PFM phase and amplitude images confirm that the applied mechanical force does not appreciably modify the ferroelectric domain configuration (Figure S7). We also exclude thickness‐dependent strain relaxation in BTO as the origin of the enhanced photocurrent (Figure S8 and Note S1), because even if strain relaxation weakens the downward polarization near the surface, the carrier‐extraction direction remains fixed by the work‐function asymmetry, and this effect is minor compared with the local drift‐field modulation induced by the mechanically generated flexoelectric field.

## 3 Mechanism of Interfacial Carrier Extraction Enhancement

**FIGURE 3:**
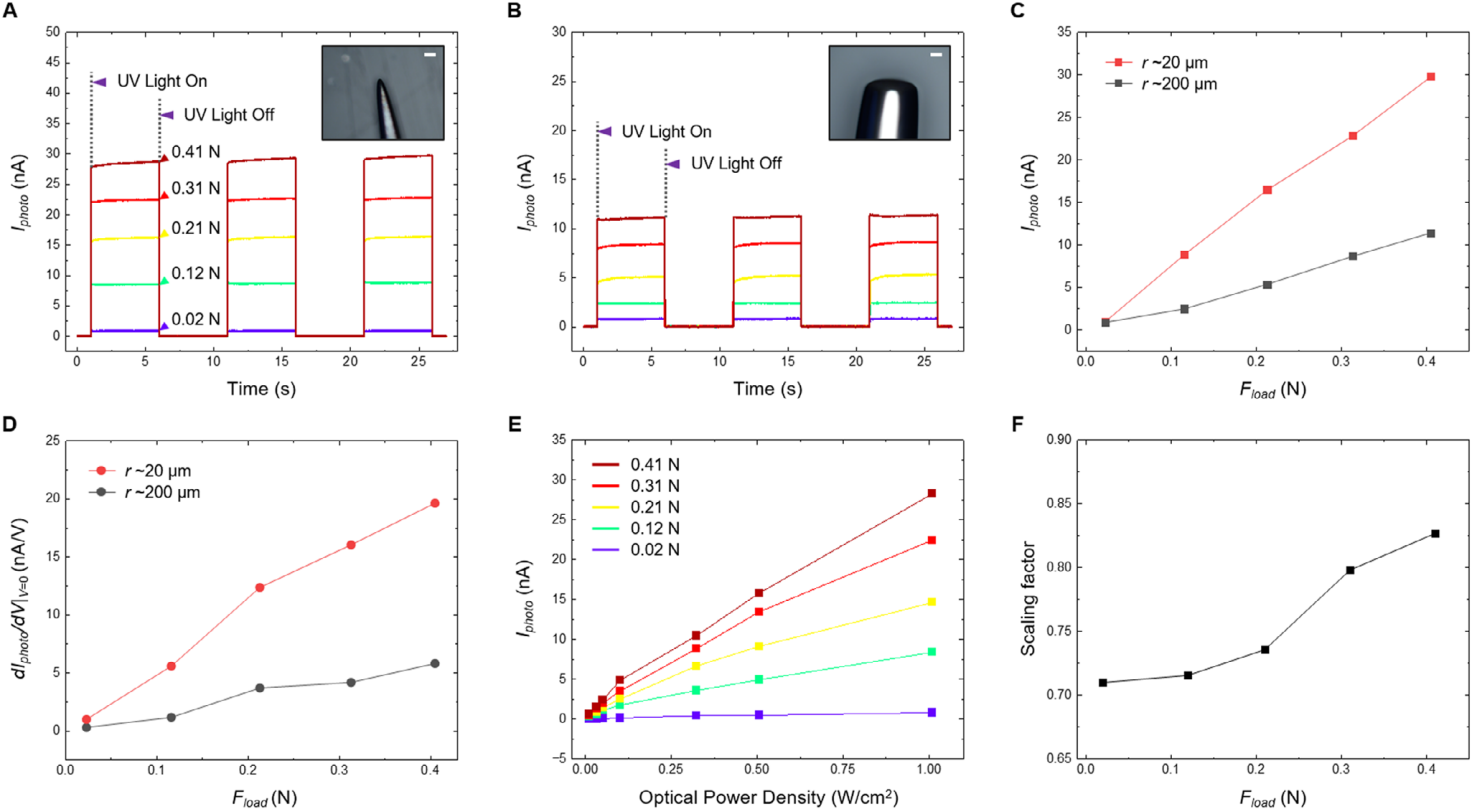
Mechanistic evidence for flexoelectric‐enhanced interfacial carrier extraction. (a,b) Time‐resolved photocurrent responses under periodic UV illumination for W‐probe tips with radius of (a) ∼20 µm and (b) ∼200 µm, respectively, measured under different *F*_load_. Insets show optical images of the respective probe tips. The white scale bar is 50 µm. (c) Photocurrent (*I*_photo_) at *V* = 0 V as a function of *F*_load_ measured by the two W‐prove tips. Although the absolute photocurrent scales with tip size, the load‐dependent enhancement trend is preserved. (d) Differential conductance (*dI*_photo_/*dV* at *V* = 0 V) as a function of *F*_load_ for both W‐probe tips, demonstrating systematic enhancement of the interfacial drift field under mechanical loading. (e) *I*_photo_ as a function of optical power (OP) density for different *F*_load_, showing progressively stronger linear scaling at higher load. (f) Extracted scaling factor from OP‐dependent *I*_photo_, monotonically increasing with *F*_load_.

To validate the enhancement of BPV performance under mechanical loading, we measured the photocurrent of the 125‐u.c.‐thick BTO sample using intensity‐modulated UV light under closed‐circuit conditions (Figure 3a). The upper right inset shows the standard W‐probe tip with a radius of ∼10 µm. The photocurrent (*I*_photo_) increases systematically with increasing *F*_load_. During both the rising and decaying processes, no significant persistent photoconductivity was observed. This behavior, consistent with the *I‐V* characteristics shown in Figure 2, confirms that mechanical loading enhances interfacial carrier extraction. To further clarify the role of the loading‐induced flexoelectric field in the enhancement of *I*_photo_, we performed similar measurements using a W‐probe tip with a much larger radius of ∼100 µm (Figure 3b). If the observed enhancement were governed by contact‐area enlargement, the larger probe would be expected to induce a higher *I*_photo_. However, although the BPV photocurrent modulation and its dependence on *F*_load_ were similarly observed, the absolute magnitude of *I*_photo_ decreased significantly. As shown in Figure 3c, *I*_photo_ remains nearly linearly proportional to *F*_load_ regardless of the tip radius, while the larger contact area results in a lower *I*_photo_. This observation clearly demonstrates that mechanical loading actively modulates the interfacial carrier extraction conditions rather than merely altering the geometric contact area. Considering the localized nature of flexoelectrically induced strain gradients, a larger probe tip is expected to distribute the strain over a broader region, thereby weakening the enhancement of the internal drift field. To further confirm that this loading‐induced modulation occurs at a structurally intact interface, we performed post‐loading cross‐sectional scanning transmission electron microscopy characterizations of the BTO/SRO/STO heterostructure used for repeated mechanical‐loading experiments (Figure S9). The mechanically contacted BTO region remained crystalline, and the buried oxide interfaces were sharp and chemically well‐defined without noticeable intermixing or degradation. These results indicate that the enhanced photocurrent does not originate from irreversible bulk damage or interfacial chemical modification, but from flexoelectric modulation of carrier extraction at the mechanically loaded BTO interface.

It is important to note that the applied mechanical force modifies the drift‐dominated carrier extraction efficiency without changing the intrinsic photogeneration characteristics of the BTO thin film. Under a small applied bias (*V*  =  *t* · *E_ext_*), the photocurrent density (*J*_photo_) can be expressed as:

(5) $$J_{photo}=en\mu \;\left(E_{drift}\right)=en\mu \left(E_{bi}+E_{ext}\right)$$

where *n* is the photo‐generated carrier density, µ is the effective mobility, and *E*_bi_ and *E*_ext_ are the built‐in and externally applied electric field, respectively. Since *E_bi_* remains constant, the small‐signal response is given by:

(6) $$\frac{dJ_{photo}}{dV}=\frac{en\mu }{t}\;\propto n\mu$$

Assuming a fixed photocarrier density under constant illumination, d*I*/d*V* at *V* = 0 V reflects the sensitivity of photocarrier transport to an external perturbation. Therefore, the nearly linear increase of d*I*/d*V*|_V = 0_ with *F*_load_, observed for both W‐probe tips (Figure 3d), confirms that mechanical loading enhances the effective interfacial transport efficiency. Indeed, d*I*/d*V*|_V = 0_ shows a much weaker dependence on *F*_load_ in the thickest (*t*_BTO_ = 500 u.c.) BTO film (Figure S10), where the flexoelectric modulation of the interfacial field becomes minimal.

Further evidence for interfacial drift enhancement and recombination suppression is obtained by analyzing the relationship between *I*_photo_ and the incident optical power density (OPD). The photocurrent typically follows a power‐law dependence [28, 29],

(7) $$I_{photo}\propto \mathrm{OP}D^{\alpha }$$

where α < 1 indicates sublinear behavior associated with recombination‐ or trap‐limited regimes. As α approaches unity, carrier extraction becomes more efficient, and recombination losses are reduced. Figure 3e shows the *I*_photo_‐OPD relationship measured at different *F*_load_. The scaling exponents extracted from power‐law fitting are shown in Figure 3f. The fitted curves are provided in Figure S11. With increasing *F*_load_, *α* increases monotonically from 0.71 to 0.83. This result quantitatively demonstrates that mechanical loading suppresses carrier recombination and associated interfacial trapping losses at the W/BTO interface, including possible losses mediated by defect‐ or interface‐related states, thereby improving carrier extraction efficiency. To examine whether the same load‐dependent enhancement is also observed when sub‐bandgap defect‐ or interface‐related excitation channels may contribute, we measured the photoresponse under 470 nm illumination (Figure S12). Although the absolute responsivity was smaller, both the photocurrent and external responsivity increased monotonically with *F*_load_, reproducing the same load‐dependent enhancement.

## 4 Theoretical Model for Flexoelectric Control of Interfacial Loss

**FIGURE 4:**
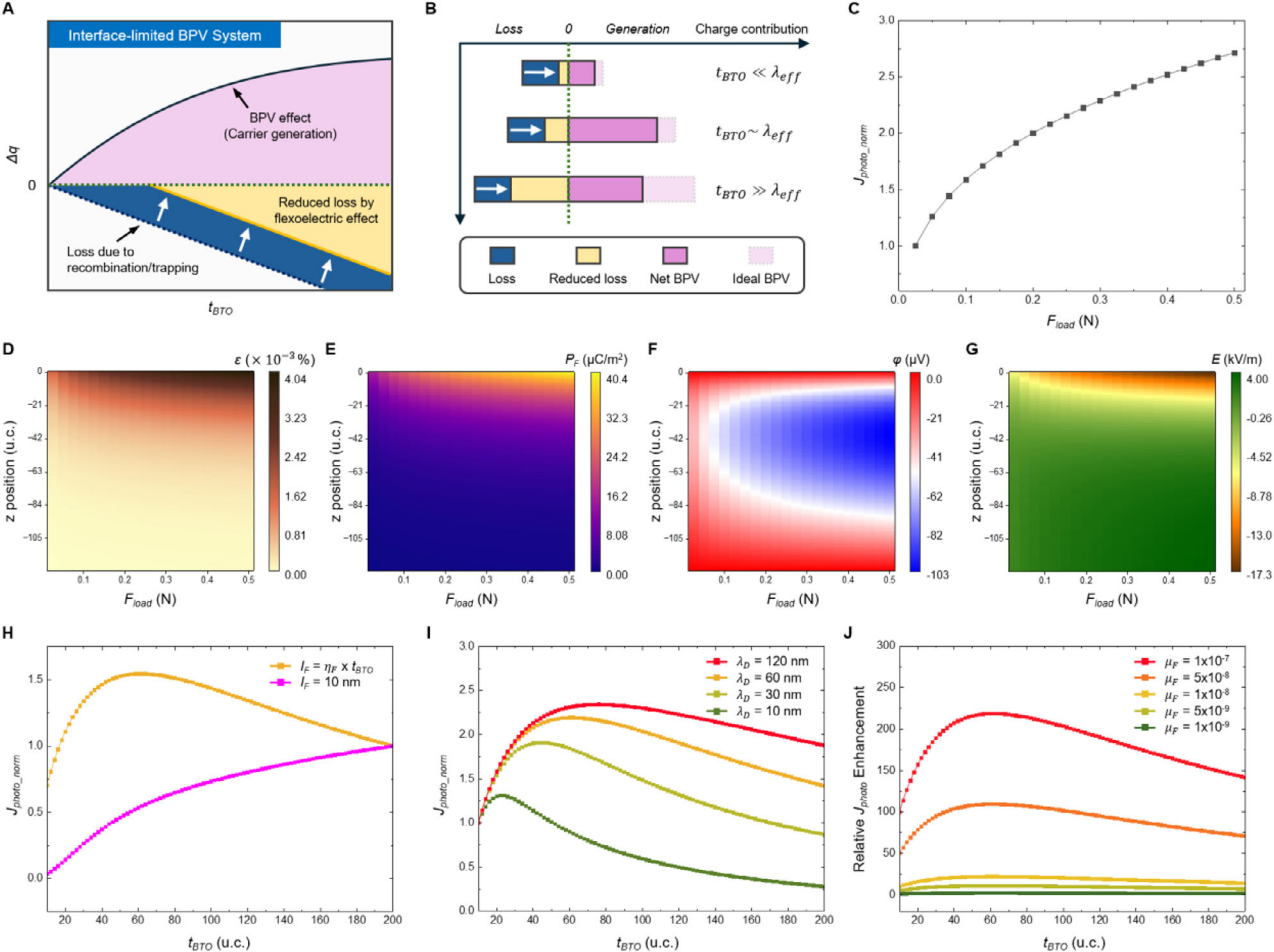
Theoretical framework for flexoelectric control of interfacial loss. (a) Conceptual illustration of carrier generation and recombination in an interface‐limited BPV film. (b) Schematic representation of carrier extraction as a function of film thickness relative to the effective carrier extraction length (*λ_eff_*). When *t_BTO_* ≪ *λ_eff_*, photocurrent is absorption‐limited, but when *t*_BTO_ ≫ *λ_eff_*, bulk recombination dominates. The optimal extraction occurs near *t_BTO_* ∼ *λ_eff_*. (c) Calculated photocurrent density (*J*_photo_) as a function of *F*_load_, reproducing the experimentally observed monotonic enhancement. To focus on the flexoelectric modulation effect, the *J*_photo_ were normalized to the value obtained at the smallest load (0.025 N). (d–g) Self‐consistent internal response of the BTO film under *F*_load_. (d) strain profile (*ε*), (e) flexoelectric polarization (*P_F_*), (f) electrostatic potential (*φ*), and (g) internal electric field (*E*). Increasing *F*_load_ enhances the interfacial drift field through flexoelectric polarization and electrostatic screening. (h) Normalized *J*_photo_ calculated with different assumptions of strain penetration length (*l*_F_), demonstrating its role as a structural control parameter. (i) Effect of Debye screening length (*λ*_D_) on thickness‐dependent photocurrent, showing that weaker screening extends flexoelectric modulation deeper into the film. (j) Relative photocurrent enhancement as a function of flexoelectric coefficient (*µ*_F_), demonstrating the nonlinear sensitivity of BPV performance to flexoelectric coupling strength.

In interface‐limited BPV systems, the photocurrent is governed by two competing processes, 1) photogeneration and 2) carrier loss due to recombination or trapping. Their expected thickness dependences are schematically illustrated in Figure 4a. Photogeneration (pink area) increases with the BTO thickness (*t*_BTO_), but exhibits a sublinear dependence due to limited optical absorption. In contrast, the recombination loss (dark blue area) approximately scales with *t*_BTO_, as the distance that photogenerated electrons must travel to reach the interface increases with thickness. Importantly, our experimental results show that this loss component can be effectively reduced via flexoelectric modulation (yellow area). This interplay naturally implies the existence of an optimal film thickness for efficient photocurrent extraction. Figure 4b further illustrates the thickness‐dependent carrier extraction in an interface‐limited BPV system. We introduce an effective carrier extraction length (*λ*_eff_), defined as the characteristic distance from the interface over which photogenerated electrons can be collected before recombination dominates. In our framework, *λ*_eff_ is not an independent parameter but emerges from the combined effects of Debye screening (*λ_D_*), field‐assisted drift transport (*µE*_drift_ *τ*), and interfacial recombination, where *τ* denotes the effective carrier lifetime. When *t_BTO_* ≪ λ_*eff*_, the photocurrent is limited by insufficient absorption volume. Conversely, when *t_BTO_* ≫ λ_*eff*_, a substantial fraction of carriers recombines in the bulk before reaching the interface. Although the loss component can be reduced by the flexoelectric modulation, bulk recombination loss becomes dominant as *t*_BTO_ increases. Therefore, the BPV efficiency is expected to be maximized when *t*_BTO_ becomes comparable to *λ*_eff_. This framework provides a unified interpretation of the photocurrent enhancement and its non‐trivial thickness dependence in BTO thin films under mechanical loading.

To quantitatively examine how flexoelectric field modulation leads to photocurrent enhancement and thickness optimization, we develop a one‐dimensional continuum model that incorporates flexoelectric polarization, electrostatic screening, and field‐assisted carrier transport. Although the actual stress field generated by tip loading is three‐dimensional, the one‐dimensional approximation is appropriate for describing the vertical flexoelectric field component that directly modulates carrier extraction in the W/BTO/SRO heterostructure. Note that mechanical loading induces a strain gradient, generating flexoelectric polarization and the associated bound charge in BTO film. The resulting electrostatic potential and electric field are calculated self‐consistently by considering charge screening inside the film. The short‐circuit photocurrent density (*J*_photo_) is evaluated from the depth‐dependent photogeneration rate (*g_photo_* (*z*)) and collection efficiency (*C*(*z*)). Photogeneration is assumed to decay exponentially with depth according to the optical absorption length (λ_*abs*_). The *C* represents the probability that a photogenerated electron reaches the electrode without recombination. This probability depends on the local electric field through the field‐assisted drift length and the effective transport distance. The total *J*_photo_ is therefore given by:

(8) $$J_{photo}=\int_{-t}^{0}g_{photo}C\;dz\;$$

A detailed description of the continuum model is provided in Note S2.

Using experimentally relevant parameters, we first examine the dependence of *J*_photo_ on the applied mechanical load. The film thickness is fixed at *t*_BTO_ = 125 u.c., and other material parameters are summarized in Note S2. To isolate the effect of *F*_load_, the calculated *J*_photo_ values were normalized to the value obtained at the smallest load (0.025 N). Figure 4c shows that the calculated *J*_photo_ increases monotonically with *F*_load_, consistent with our experimental observations. Notably, the sublinear dependence indicates that the enhancement does not originate simply from lattice deformation, but rather from the strain‐gradient‐induced interfacial modification.

To elucidate the physical origin of the load‐dependent enhancement of *J*_photo_, we also examine the internal electrostatic response of the BTO film. The out‐of‐plane strain profile ε(*z*) as a function of *F*_load_ is shown in Figure 4d. Mechanical loading generates a pronounced strain gradient near the top interface. This strain gradient induces flexoelectric polarization *P_F_*, leading to the formation of bound charges near the interface (Figure 4e). The electrostatic equilibrium is subsequently reconfigured through the charge screening of these bound charges. This reconfiguration is reflected in the self‐consistently calculated electrostatic potential profiles shown in Figure 4f. As *F*_load_ increases, the potential gradient near the interface becomes progressively enhanced, reflecting a modification of the interfacial band bending. Although the potential redistribution becomes more extended at higher *F*_load_, the potential gradient remains largest near the interface, indicating that flexoelectric modulation modifies the interfacial electric field rather than producing a uniform bulk shift. Figure 4g shows the corresponding electric field as a function of *F*_load_. The drift field near the top interface is markedly enhanced with increasing *F*_load_. This enhanced field effectively shortens the residence time of photogenerated electrons in the recombination‐active region, thereby increasing the probability that carriers are extracted to the electrode before recombination occurs. As a result, the effective carrier extraction length (*λ_eff_*) increases with *F*_load_, in quantitative agreement with the framework introduced in Figure 4b. In this sense, the flexoelectric strain gradient serves as a control parameter for the interfacial electrostatic screening length, providing a direct mechanism for suppressing interfacial loss.

In addition, our model provides several important insights into interface‐limited BPV systems. First, we clarify the role of the strain penetration length (*l*_F_). Figure 4h shows *J*_photo_ as a function of *t*_BTO_ under different assumptions for *l*_F_. When *l*_F_ is assumed to scale proportionally with *t*_BTO_ (orange squares), the nonlinear thickness dependence is well reproduced. In contrast, when *l*_F_ is set as a constant (pink squares), the calculated thickness dependence deviates significantly from experiment. This indicates that the strain gradient generated by mechanical loading is not confined to the immediate interfacial region, but scales with the film thickness. Therefore, the strain penetration length emerges as an effective structural length scale that critically governs the efficiency of flexoelectric modulation. Next, we examine the influence of the Debye screening length (λ_*D*_), which proves to be another key parameter controlling BPV performance. As shown in Figure 4i, increasing λ_*D*_ shifts the optimal *t*_BTO_ to larger thicknesses and elevates *J*_photo_ across the thickness range. This is because a longer screening length allows the flexoelectric modulation to extend deeper into the film. Accordingly, materials with lower carrier density or weaker screening can transmit flexoelectric‐induced electrostatic modulation more effectively across the film thickness. Thus, the Debye length is not merely an intrinsic electrical parameter, but a critical materials‐selection metric for optimizing thin‐film BPV systems. Finally, we evaluated the sensitivity of the calculated BPV response to the effective flexoelectric coefficient (µ_*F*_) (Figure 4j). The µ_*F*_ represents an effective coefficient under the mechanical loading geometry. The calculated *J*_photo_ enhancement increases nonlinearly as µ_*F*_ varies from 10^−9^ to 10^−7^ C/m, because a larger µ_*F*_ generates stronger strain‐gradient‐induced polarization and interfacial electric‐field modulation. This sensitivity analysis confirms that the magnitude of the enhancement depends on µ_*F*_, while the proposed flexoelectric modulation mechanism remains valid over the examined coefficient range. Collectively, our modeling results establish a strain‐mediated electrostatic framework in which structural, electrostatic, and materials parameters cooperatively determine carrier extraction and interfacial loss in interface‐limited BPV systems.

## 5 Discussion

In summary, we demonstrate that interfacial loss in thin‐film BPV systems can be effectively suppressed through mechanically induced flexoelectric strain gradients. By combining systematic loading experiments with self‐consistent electrostatic modeling, we show that strain gradients reconfigure interfacial electrostatics, enhance the local drift field, and thereby accelerate carrier extraction at the interface. This flexoelectric modulation substantially enhances photocurrent by mitigating interfacial recombination, and also establishes an optimal thickness for efficient photocurrent extraction. Beyond reproducing the experimental trends, our modeling framework also reveals that structural (*l_F_*), electrostatic (λ_*D*_), and materials‐specific (µ_*F*_) parameters cooperatively govern carrier extraction in interface‐limited BPV systems. These results establish strain‐gradient‐driven electrostatic control as a physically grounded strategy for interfacial loss engineering. Finally, we emphasize that flexoelectricity can influence photovoltaic behavior through multiple physical pathways. Recent studies have shown that flexoelectric polarization itself can generate large photocurrents [30, 31, 32, 33, 34], even in nominally non‐ferroelectric systems [35]. By contrast, our results reveal a distinct regime in which flexoelectricity enhances photovoltaic output not by creating additional photogeneration, but by suppressing interfacial carrier loss in thin‐film BPV systems. Interfacial loss control thus emerges as a key parameter for translating intrinsic photogeneration into measurable photocurrent. The concurrent optimization of intrinsic photogeneration and interfacial loss control may ultimately provide a viable route toward the practical realization of high‐performance thin‐film BPV devices.

## 6 Experimental Section

### 6.1 BTO Thin Film Synthesis

The BTO and SRO thin films were epitaxially grown on STO (001) substrates by PLD. The as‐received STO substrates (Shinkosha) were first etched in buffered hydrofluoric acid and subsequently annealed at 900°C for 6 h to obtain a TiO_2_‐terminated surface. After thermal treatment, SRO and BTO films were grown in‐situ on the STO substrates. The thickness of the SRO layer was fixed at 15 nm, while the BTO thickness (*t*_BTO_) was varied as described in the main text. The deposition rates of SRO and BTO were calibrated by X‐ray reflectivity (XRR) measurements. For SRO growth, a ceramic SRO target was ablated using a KrF excimer laser (λ = 248 nm) with a repetition rate of 3 Hz and a laser fluence of ∼0.86 J/cm^2^. During SRO deposition, the substrate temperature and oxygen partial pressure were maintained at 660°C and 50 mTorr, respectively. The growth conditions were then adjusted to 630°C and 75 mTorr for BTO deposition. The repetition rate and laser fluence for BTO growth were 3 Hz and ∼0.76 J/cm^2^, respectively. After deposition, the BTO/SRO/STO heterostructures were cooled to room temperature in an oxygen atmosphere. Bottom electrical contacts were made using commercial indium without conventional lithographic lift‐off processes.

### 6.2 Photocurrent Measurements Under Mechanical Loading

For electrical measurements, the indium at the corner of samples were used as a bottom contact. We used a commercial W‐probe tip to contact directly onto BTO films to measure output current. The W‐probe tip was positioned vertically to minimize lateral sliding on the film surface, allowing it to function as an electrical contact and also as a mechanical loading source simultaneously. The applied mechanical load was controlled using a custom‐built tuning knob in combination with a micromanipulator. For reproducible loading conditions, the knob was adjusted in discrete click units. The applied force corresponding to each rotational increment of the knob was calibrated, enabling systematic and quantitative control of the loading force. Under controlled mechanical loading, a UV laser (λ = 405 nm, optical power = 97.4 mW) was focused onto the center of the sample to generate photocurrent. The photocurrent was then measured using a Keithley 4200A‐SCS (Tektronix). All measurements were performed in a completely dark and electrically shielded chamber. The optical power of UV light was controlled by commercial Neutral Density (ND) filters (Edmund optics).

### 6.3 Sample Temperature Measurements

The temperature of BTO sample was measured by a thermal imaging camera (CSIR‐10, Acuba) with and without UV light. The distance between the camera and the sample was fixed during the measurement. No external lamp or power supplier was operated during the measurements.

### 6.4 Annular Dark‐Field Scanning Transmission Electron Microscopy (ADF‐STEM) and Energy‐Dispersive X‐Ray Spectroscopy (EDS) Measurements

A cross‐sectional BTO/SRO/STO heterostructure sample was prepared using a focused ion beam (FIB) machine (Helios G5, Thermo Fisher Scientific) with a gallium ion source. The FIB was operated at an accelerating voltage of 30 kV. Prior to FIB milling, a thin carbon layer around 25 nm thickness was deposited to minimize ion beam‐induced specimen damage. Both ADF‐STEM and EDS measurements were conducted using a double Cs‐corrected TEM (Spectra Ultra, Thermo Fisher Scientific) operated at 300 kV, with a beam convergence semi‐angle of 21.2 mrad. For ADF‐STEM measurements, images were acquired with a frame size of 4096 × 4096 pixels, a pixel size of 8.4 pm, and a dwell time of 0.5 µs. The images in Figure S9a,b were acquired at screen currents of 30 and 70 pA, respectively, with corresponding ADF detector inner and outer collection angles of 49 and 200 mrad for Figure S9a and 63 and 200 mrad for Figure S9b. EDS spectra were obtained with a pixel size of 382.3 pm, a dwell time of 5.0 µs, and a screen current of 100 pA over 20 frames. Elemental EDS maps were extracted from the Kα lines for O, Ti, and Sr and the Lα lines for Ru and Ba. Gaussian smoothing with a standard deviation of 5 pixels was subsequently applied to the maps.

### 6.5 Self‐Consistent Electrostatic Modeling

A one‐dimensional continuum model was developed to describe the flexoelectric modulation of interfacial electrostatics in BTO thin films. The model incorporates strain‐gradient‐induced flexoelectric polarization, electrostatic screening governed by the Debye length, and field‐assisted carrier drift transport. The potential and electric field were obtained self‐consistently by solving Poisson's equation with bound charge contributions. The *J*_photo_ was evaluated by integrating the depth‐dependent photogeneration rate and carrier collection probability across the film thickness. Detailed equations, boundary conditions, and parameter values are provided in Note S2.

## Author Contributions

**Minwoo Jang**: investigation, formal analysis, writing – original draft. **Hyungwoo Lee**: conceptualization, formal analysis, supervision, funding acquisition, writing – original draft, writing – review and editing. **Jaewhan Oh**: methodology, investigation, validation, writing – review and editing. **Hyunkyu Lim**: investigation, formal analysis, writing – original draft. **Yongsoo Yang**: methodology, investigation, validation, formal analysis, writing – review and editing. **Sanghoon Yeom**: investigation, formal analysis, writing – original draft.

## Conflicts of Interest

The authors declare no conflicts of interest.

## Supporting information




**Supporting File**: advs76848‐sup‐0001‐SuppMat.docx.

## Data Availability

The data that support the findings of this study are available from the corresponding author upon reasonable request.
