# Supplementary material for: Flexoelectric Suppression of Interfacial Carrier Loss in BaTiO3 Thin‐Film Bulk Photovoltaic Systems
Source: Adv Sci (Weinh). 2026 Jul 29:e76848. Online ahead of print. doi: 10.1002/advs.76848 (PMC13418503; doi:10.1002/advs.76848)
Supplement: Supplementary file 1 — Supporting File: advs76848‐sup‐0001‐SuppMat.docx. [file ADVS-9999-e76848-s001.docx]

Supporting Information

Flexoelectric Suppression of Interfacial Carrier Loss in BaTiO_3_ Thin-Film Bulk Photovoltaic Systems

Minwoo Jang, Hyunkyu Lim, Sanghoon Yeom, Jaewhan Oh, Yongsoo Yang, and Hyungwoo Lee*

M. Jang, H. Lim, S. Yeom

Department of Energy Systems Research, Ajou University, Suwon, Republic of Korea

J. Oh

Department of Physics, Korea Advanced Institute of Science and Technology (KAIST), Daejeon, 34141, Republic of Korea

Y. Yang

Department of Physics, Korea Advanced Institute of Science and Technology (KAIST), Daejeon, 34141, Republic of Korea

Graduate School of Semiconductor Technology, School of Electrical Engineering, Korea Advanced Institute of Science and Technology (KAIST), Daejeon, 34141, Republic of Korea

H. Lee

Department of Physics, Ajou University, Suwon, Republic of Korea

Department of Energy Systems Research, Ajou University, Suwon, Republic of Korea

E-mail: [hyungwoo@ajou.ac.kr](mailto:hyungwoo@ajou.ac.kr)

**
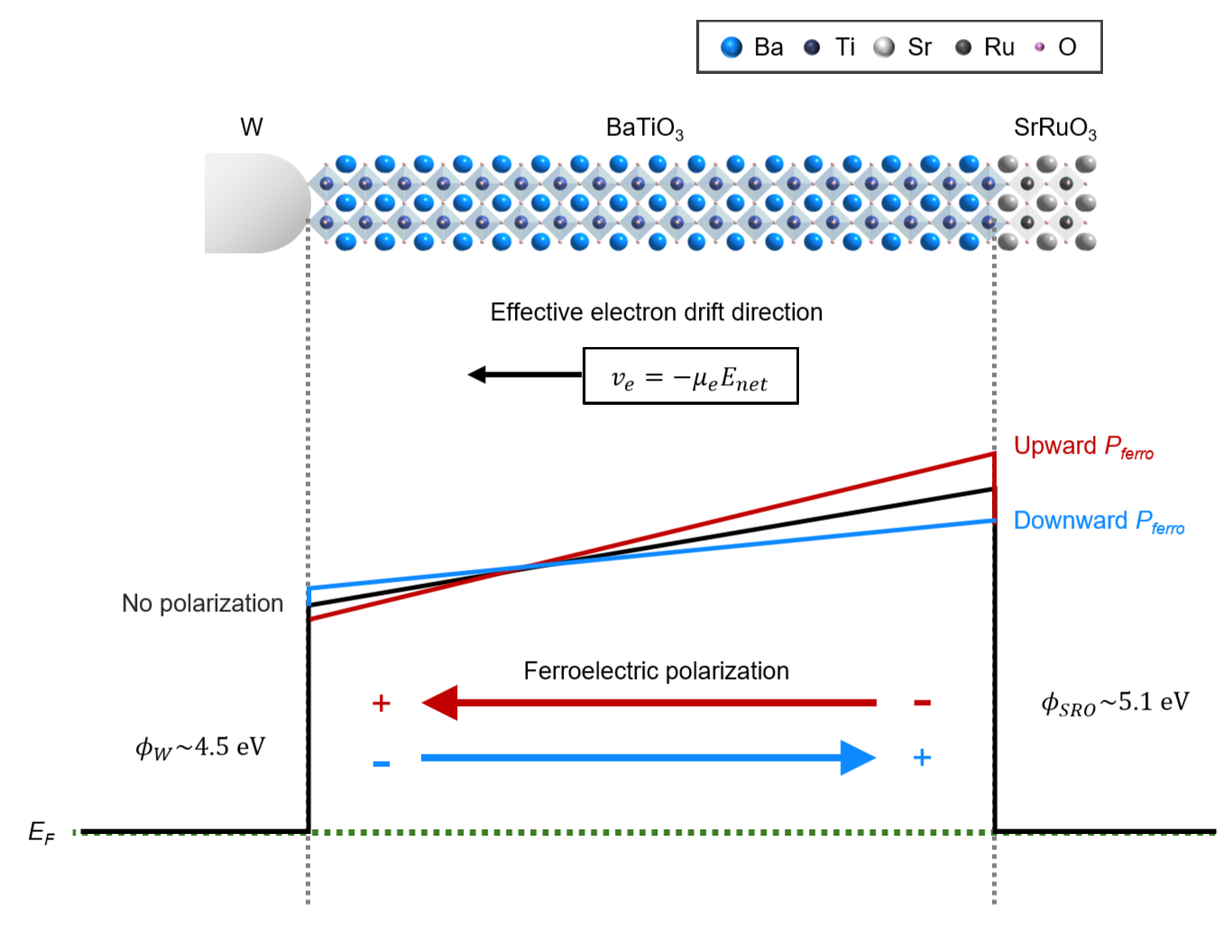
**

**Figure S1. Drift direction of photoelectrons in W/BaTiO_3_/SrRuO_3_ (W/BTO/SRO) heterostructures.** In W/BTO/SRO heterostructures, the conduction band of the BTO layer is strongly tilted due to (1) intrinsic work-function asymmetry between the electrodes and (2) the ferroelectric polarization of BTO (*P_ferro_*). Considering the work functions of W ($\phi_{W}\sim4.5 \mathrm{eV}$) and SRO ($\phi_{SRO}\sim5.1 \mathrm{eV}$), the electrode-driven band offset is estimated to be ~0.6 eV. In comparison, the electrostatic modulation arising from ferroelectric polarization is relatively smaller. Taking a typical ferroelectric polarization of BTO thin films ($P_{ferro}\approx20-30 \mu C \mathrm{cm}^{-2}$), complete polarization reversal would correspond to $\Delta P_{ferro}\approx40-60 \mu C \mathrm{cm}^{-2}$. In the absence of screening, the depolarization field ($E_{D}={\Delta P_{ferro}}/{\varepsilon_{0}\varepsilon_{r}}$) is approximately 2.8 MV/cm, where the vacuum permittivity ($\varepsilon_{0}=8.85\times{10}^{-12}$ F/m) and the relative permittivity of BTO ($\varepsilon_{r}=200$) are assumed. However, when ferroelectric films are directly contacted with metallic electrodes, the depolarization field is largely screened, and typically only ~10% of $E_{D}$ (i.e., ~0.28 MV cm^-2^) survives. For a 125-unit-cell (u.c.)-thick BTO film, this corresponds to a potential modulation of ~0.14 V induced by *P_ferro_*. Therefore, as shown in Figure S1, photoelectrons naturally drift toward the upper W electrode. Even when *P_ferro_* is switched up and down, the polarization-induced modulation is insufficient to overcome the electrode-driven band offset and reverse the electron drift direction. Accordingly, unless otherwise specified, we focus on the downward *P_ferro_* configuration, where the photocurrent was maximized. When considering only the drift field, the upward $P_{ferro}$ appears more favorable for carrier extraction than the downward one. However, the steeper potential gradient also increases the effective carrier density near the W/BTO interface, thereby enhancing interfacial recombination. In contrast, although the downward $P_{ferro}$ leads to a slightly weaker drift field, the reduced interfacial loss leads to a higher net photocurrent. The effect of ferroelectric polarization on photocurrent will be discussed more in detail later.


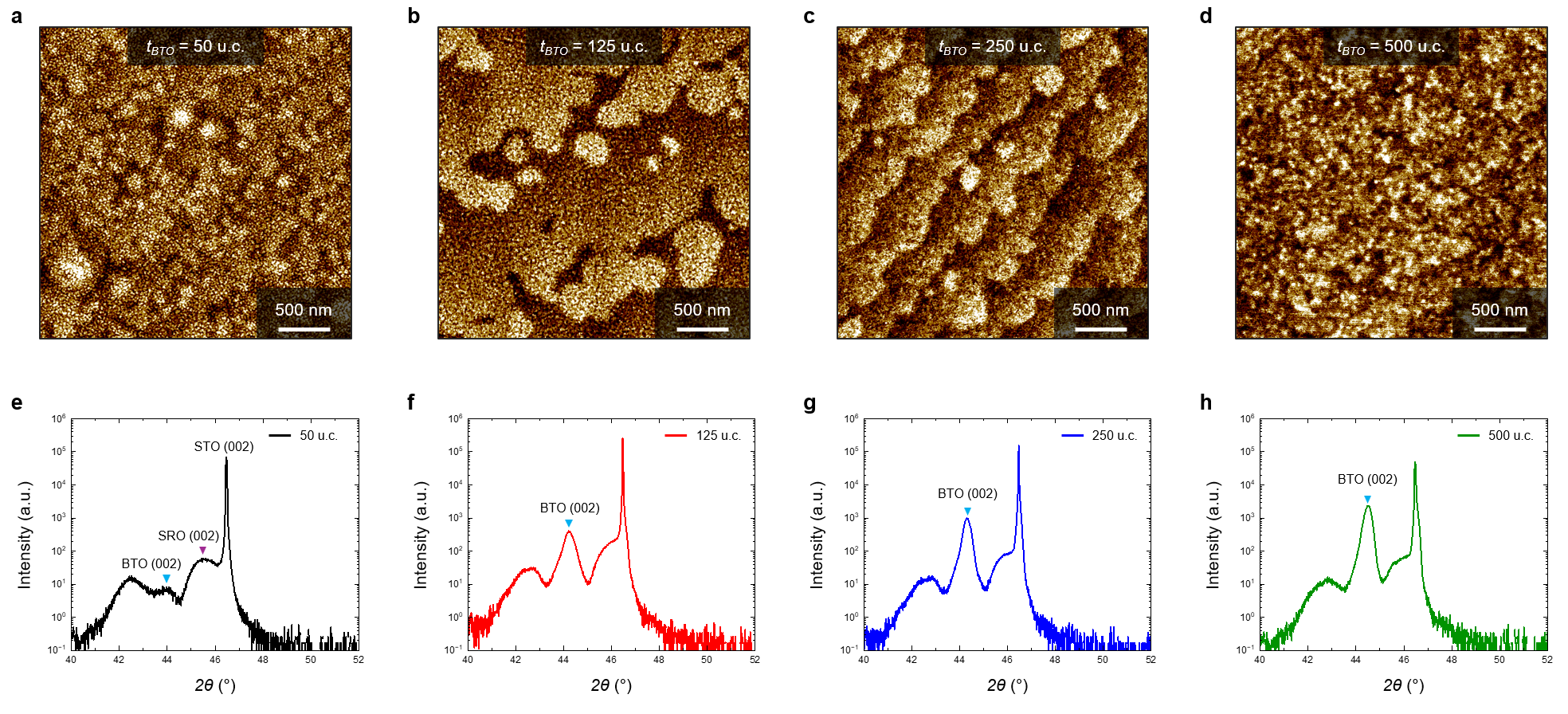


**Figure S2. Structural characterizations of BTO/SRO/STO heterostructures.** (a-d) Atomic force microscopy (AFM) images of as-grown BTO/SRO/STO samples with BTO thicknesses (*t_BTO_*) of (a) 50 u.c., (b) 125 u.c., (c) 250 u.c., and (d) 500 u.c. The step-and-terrace structure of the thermally treated STO (001) substrates is well preserved on the BTO thin films, indicating their high quality. (e-h) X-ray diffraction (XRD) *θ*-2*θ* scans of the corresponding samples. While thicker BTO films show slight strain relaxation, all BTO films exhibit a single, well-defined (002) reflection, confirming their high crystallinity.


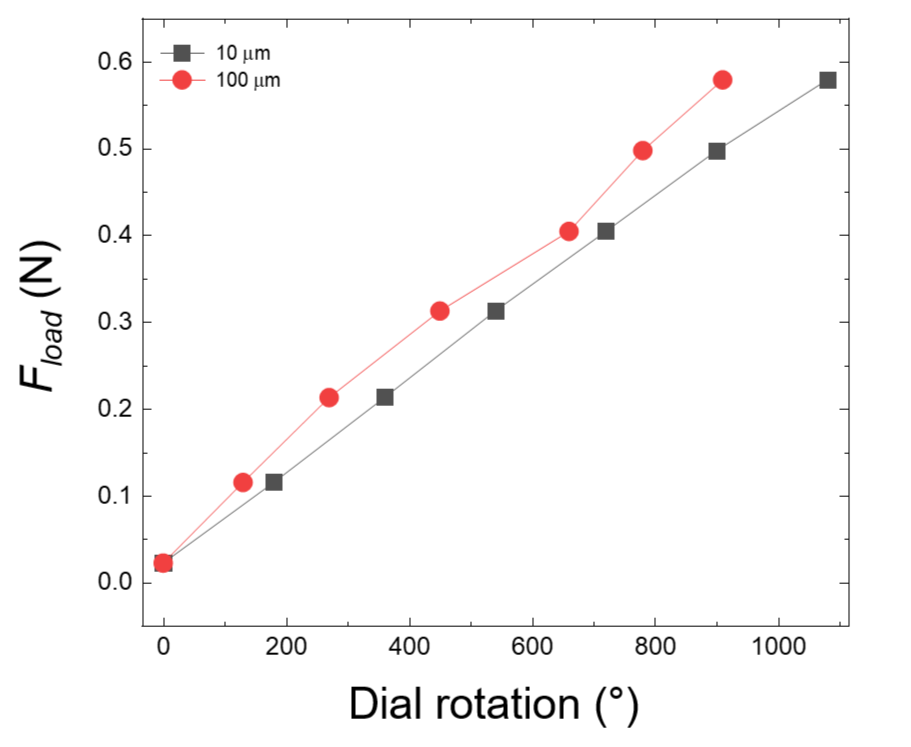


**Figure S3. Loading force calibration.** The loading force (*F_load_*) was controlled using a custom-built tuning knob. To ensure reproducible force application during repeated measurements, the knob was adjusted in discrete click steps. Figure S3 shows the measured dial rotation angle corresponding to each click and the resulting *F_load_*. Although the same tuning knob and micromanipulator were used throughout the experiments, separate calibrations were carried out for two probe tips with different radii to eliminate potential artifacts arising from differences in contact area.


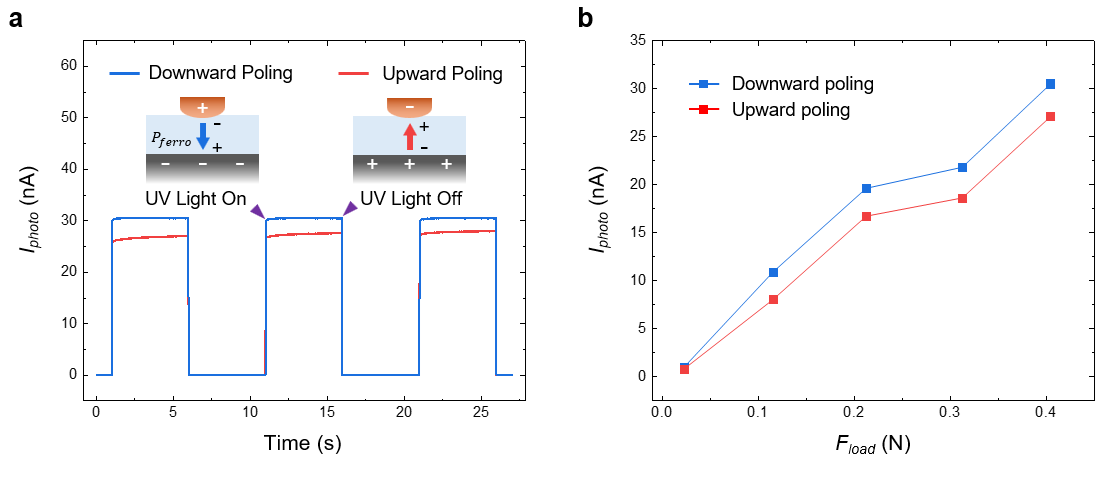


**Figure S4. Ferroelectric polarization (*P_ferro_*) effect on photocurrent.** (a) Time-resolved photocurrent responses of the BTO thin film (*t_BTO_* = 125 u.c.) under periodic UV illumination for downward (blue) and upward (red) polarization. The inset schematics illustrate the corresponding poling configurations. Before the photocurrent measurements, we applied -2 V (+2 V) at the bottom SRO electrode for ~1 s to set the downward (upward) polarization state. The photocurrent is consistently higher for the downward *P_ferro_* configuration. (b) Photocurrent (*I_photo_*) as a function of the applied loading force (*F_load_*) for the two polarization directions. The two configurations exhibit nearly identical *F_load_*-dependent behaviors, indicating that the dominant driving force for electron drift originates from the intrinsic band offset and the associated built-in field in the W/BTO/SRO heterostructure, rather than *P_ferro_* itself. Although the upward *P_ferro_* nominally produces a slightly larger band slope near the interface (see Figure S1), this difference is minor because the overall band tilt is dominated by the work-function asymmetry between the W and SRO electrodes. Instead, the observed photocurrent asymmetry is attributed to the interplay between flexoelectric field amplification and trap-assisted recombination near the interface. In the upward-*P_ferro_* state, stronger band bending leads to a larger accumulation of photoelectrons in the trap-rich interfacial region (i.e., W/BTO interface), increasing recombination loss. In contrast, the downward-*P_ferro_* state results in a smaller interfacial carrier population and reduced trap-assisted recombination, allowing more photogenerated electrons to be extracted despite the slightly weaker nominal drift field.


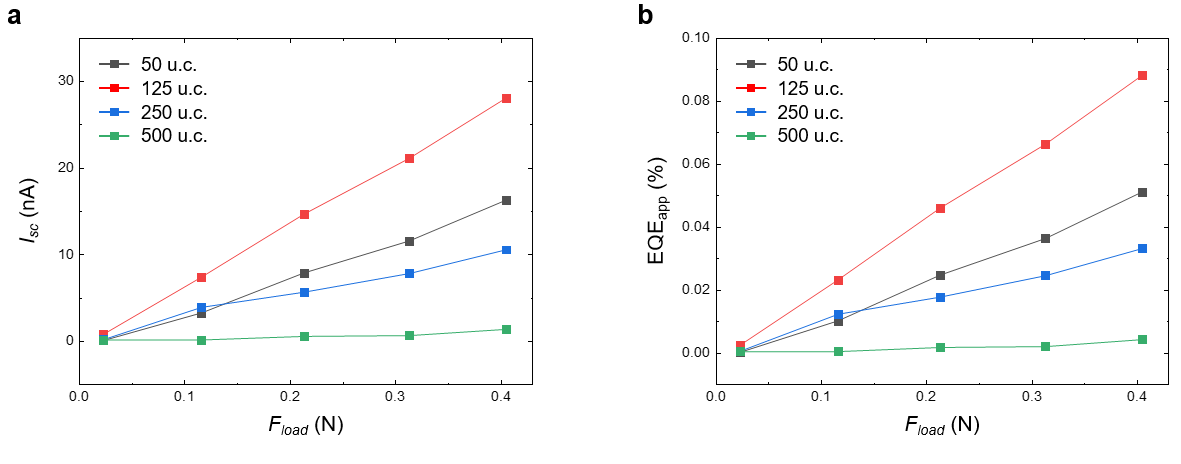


**Figure S5. Thickness-dependent short-circuit current and derived EQE under mechanical loading (*F_load_*).** (a) Short-circuit current (*I_sc_*) measured as a function of applied *F_load_* for BTO thin films with thicknesses of 50, 125, 250, and 500 u.c. All the *I_sc_* values were obtained under the identical optical conditions described in main text. (b) External quantum efficiency (EQE) calculated from the measured *I_sc_* values shown in (a). The apparent EQE (EQE_app_) is calculated using the total incident optical power. Its relationship with responsivity (*R*) can be written as ${EQE}_{app}=R\cdot\frac{hc}{q\lambda}$, where *h*, *c*, *q,* and $\lambda$ are Planck constant, the speed of light, unit charge, and the wavelength of light (405 nm), respectively. The consistent *F_load_*-dependent enhancement across thicknesses confirms that the mechanical modulation effect is robust and independent of the chosen performance metric.


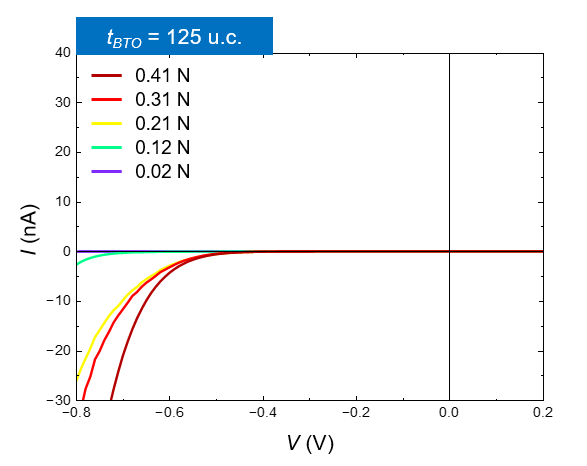


**Figure S6. Dark I-V characteristics under mechanical loading.** Dark I-V characteristics of the BTO thin film (*t_BTO_* = 125 u.c.) measured under different *F_load_*_._ While the reverse-bias current increases with increasing *F_load_*, the forward-bias current and the overall diode-like behaviors are almost identical. The increase in reverse-bias current represents the field-assisted leakage transport, where the *F_load_*-induced enhancement of interfacial transport efficiency can facilitate carrier transport through the interface. In contrast, the forward-bias current is governed by carrier injection from the electrode, and therefore remains largely unchanged by mechanical loading.

**
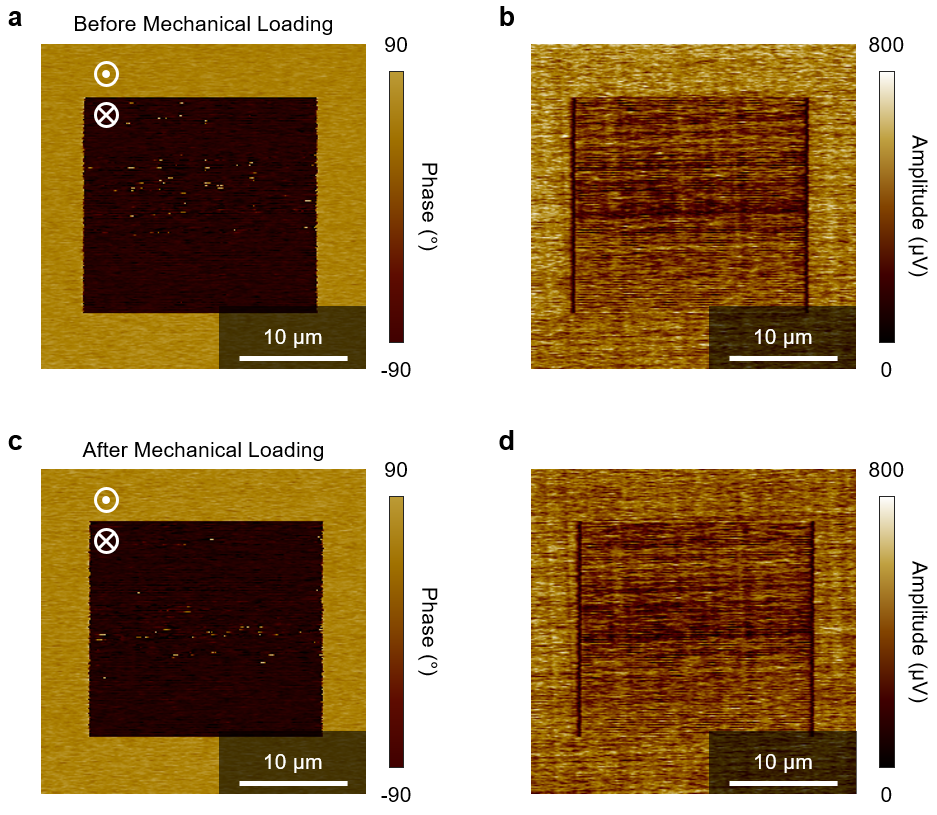
**

**Figure S7. Effect of mechanical loading on the domain configuration in the BTO thin film.** (a, b) Piezoresponse force microscopy (PFM) (a) phase and (b) amplitude images, respectively, measured immediately after downward poling of the BTO sample (*t* = 125 u.c.) by applying -2 V to the bottom contact of the sample. (c,d) PFM (c) phase and (d) amplitude images, respectively, acquired from the same region after the sample was removed from the AFM, mechanically loaded under the same conditions used in the loading experiment, and then re-mounted for PFM imaging. The nearly unchanged phase and amplitude contrast before and after mechanical loading indicates that the loading process did not induce noticeable ferroelectric domain reconfiguration in the poled region. PFM imaging was performed using an AFM system (NX10, Park Systems). In PFM mode, an AC bias of 3.5 V at 20 kHz was applied during polarization imaging. All measurements were carried out using Cr/Pt-coated tips (ElectriMulti75-G, Budget Sensors) under controlled environmental conditions of ~22.1 °C and ~25% relative humidity. Note that the PFM phase and amplitude images before and after mechanical loading are nearly identical, showing no appreciable change in the domain pattern. These results confirm that the applied mechanical loading does not induce significant domain reconfiguration under our experimental conditions. Therefore, the observed photocurrent enhancement cannot be attributed to mechanically induced polarization switching or domain rearrangement.


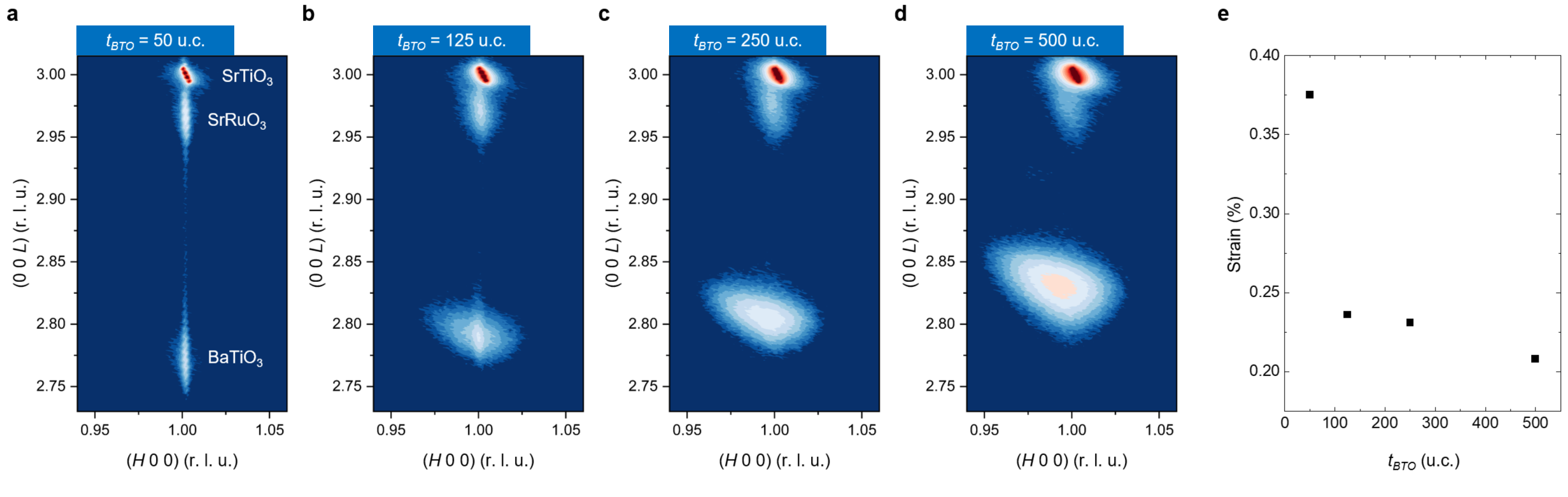


**Figure S8. Thickness-dependent structural relaxation of BTO films.** (a-d) Reciprocal-space maps of BTO/SRO/STO(001) heterostructures with BTO thicknesses (*t_BTO_*) of 50, 125, 250, and 500 u.c., respectively. The BTO diffraction feature becomes progressively shifted and broadened with increasing *t_BTO_*, indicating partial strain relaxation. (e) Strain values estimated by the Stokes-Wilson method from the *θ*-2*θ* scans shown in Fig. S2. The detailed method is described in Supplementary Note S1. The estimated strain decreases with increasing BTO thickness, further supporting gradual strain relaxation in thicker BTO films. Although this relaxation may slightly reduce the overall polarization magnitude compared with ideally coherent BTO films, the films retain ferroelectric characteristics, in agreement with the PFM measurements (Figure S7). Importantly, this thickness-dependent relaxation represents a static structural effect and therefore cannot account for the mechanically induced, loading-force-dependent photocurrent enhancement observed at a fixed film thickness. In addition, relaxation-induced reduction of the polarization magnitude would be expected to weaken, rather than enhance, polarization-assisted carrier extraction. As discussed in Fig. S1, the carrier-extraction efficiency is therefore mainly set by the intrinsic work-function asymmetry and is further enhanced by mechanically induced flexoelectric modulation. The strain relaxation may only provide a secondary contribution to the baseline polarization state.

**
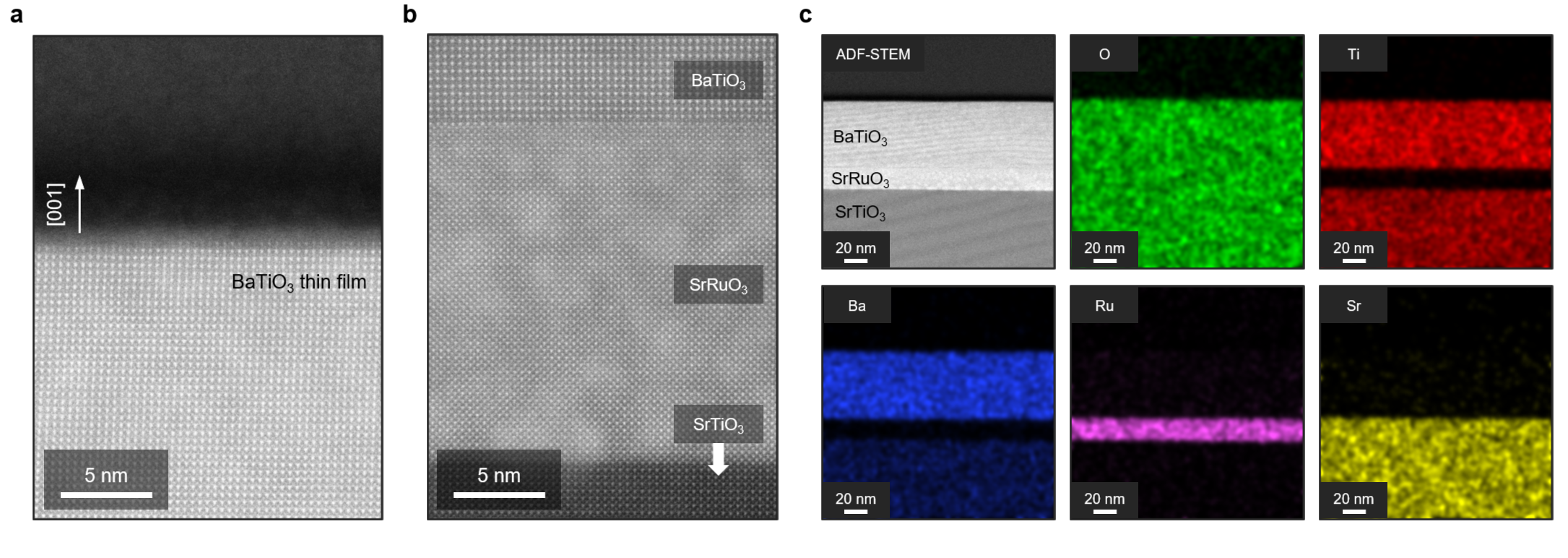
**

**Figure S9. Post-loading Cross-sectional scanning transmission electron microscopy (STEM) characterizations of the mechanically loaded BTO/SRO/STO heterostructure.** (a) Cross-sectional Annular dark-field (ADF)-STEM image of the BTO surface region after repeated mechanical-loading experiments. The TEM specimen was prepared from the mechanically tested sample after detaching the W contact, allowing the BTO side of the loaded W/BTO interface to be examined. The BTO film retains a crystalline lattice structure without observable cracking, amorphization, or mechanically induced structural degradation. (b) Cross-sectional ADF-STEM image of the buried BTO/SRO and SRO/STO interfaces. Both oxide interfaces remain sharp and well defined after the loading experiments, indicating that repetitive mechanical loading does not induce detectable interfacial disruption or delamination. (c) An ADF-STEM image and corresponding STEM-energy-dispersive X-ray spectroscopy (EDS) elemental maps of O, Ti, Ba, Ru, and Sr. The elemental distributions clearly resolve the BTO layer, SRO bottom electrode, and STO substrate, with no noticeable cation interdiffusion or intermixing. These results confirm that the loading-dependent photocurrent enhancement was measured in a structurally intact oxide heterostructure and therefore is not caused by irreversible mechanical damage, interfacial degradation, or chemically induced extrinsic effects. Rather, they support that the enhanced photocurrent originates from flexoelectric modulation of carrier extraction at the mechanically loaded BTO interface.


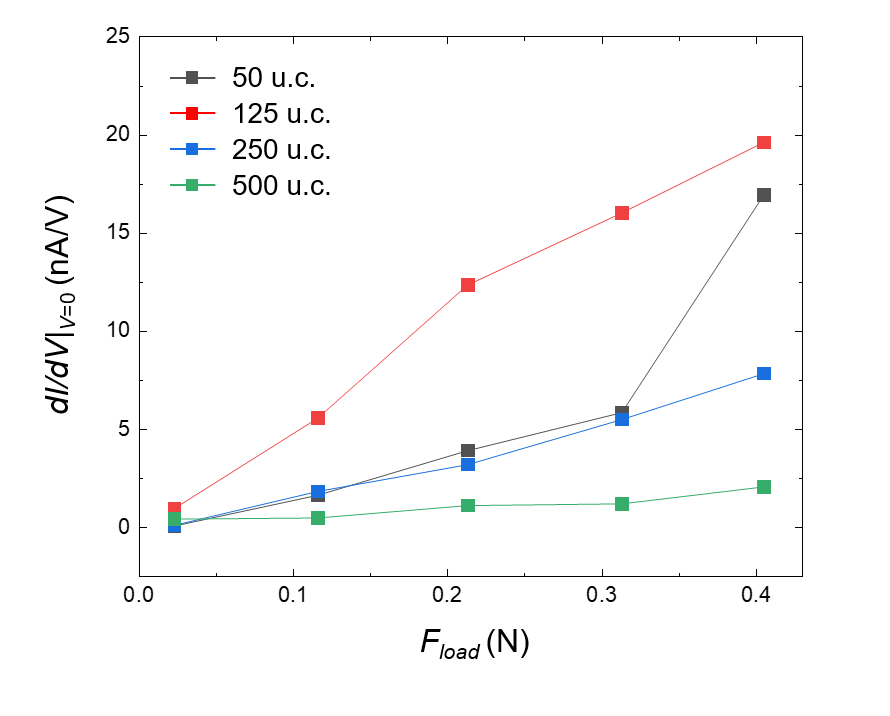


**Figure S10. Thickness (*t_BTO_*) dependence of loading-induced transport modulation.** Differential conductance (*dI_photo_*/*dV* at *V* = 0 V) as a function of *F_load_* measured at BTO films with thicknesses (*t_BTO_*) of 50, 125, 250, and 500 u.c. The sensitivity of d*I*/d*V*|_V=0_ to *F_load_* varies with *t_BTO_* and becomes significantly weaker for the thicker BTO films (*t_BTO_* of 250 u.c. and 500 u.c.). This trend is consistent with the reduced flexoelectric modulation of the interfacial electric field in thicker films.


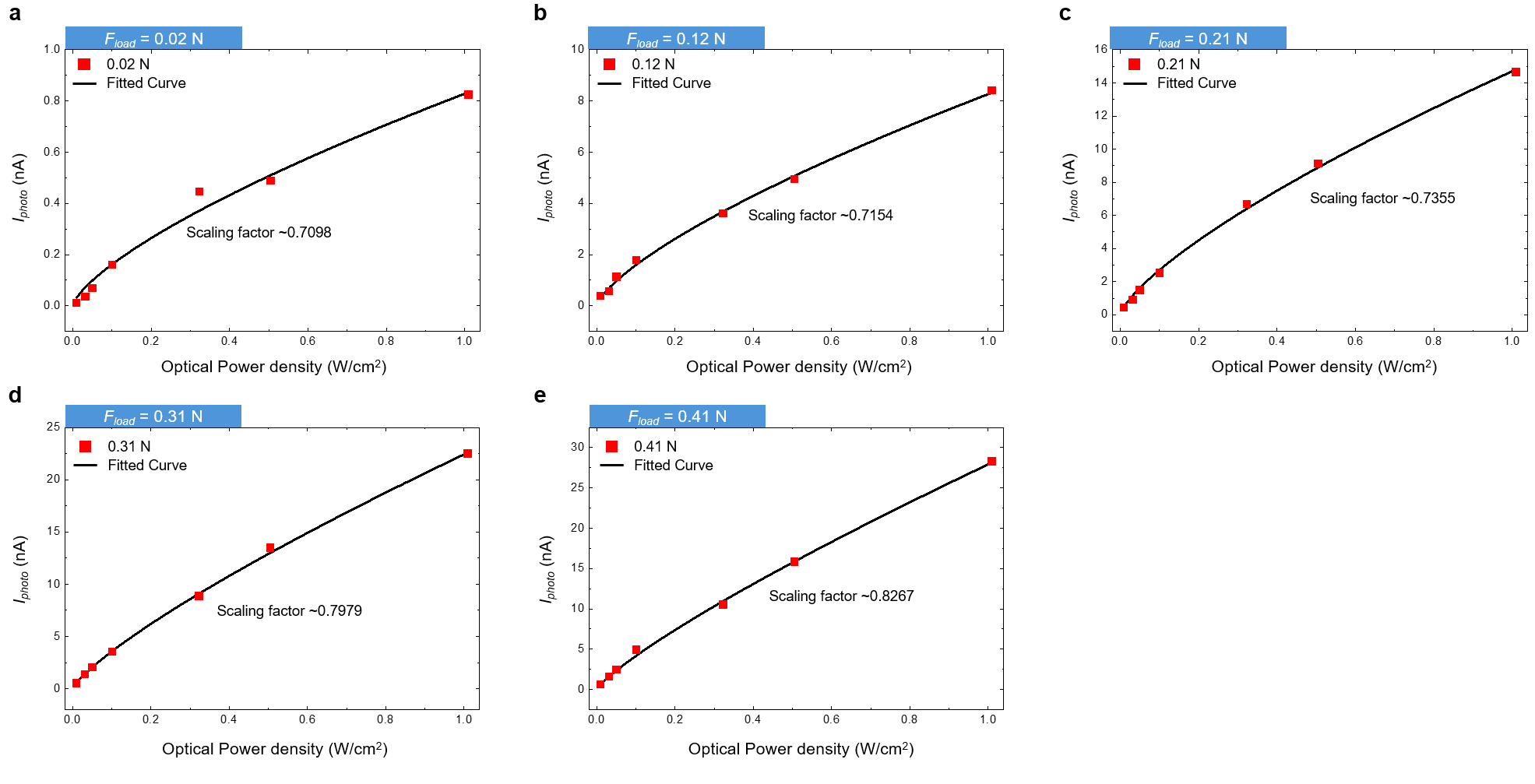


**Figure S11. *F_load_*-dependent scaling behavior of photocurrent (*I_photo_*) as a function of optical power density (OPD).** (a-e) *I_photo_* plotted against OPD under *F_load_* of (a) 0.02 N, (b) 0.12 N, (c) 0.21 N, (d) 0.31 N, and (e) 0.41 N. The OPD of the incident UV light was controlled using commercial neutral density filters. All datasets were well fitted by a power law relationship, $I_{photo}=A\cdot{OPD}^{\alpha}$, where *A* is a proportional coefficient. The extracted scaling factor ($\alpha$) for each load condition is indicated next to the corresponding fitting curve.


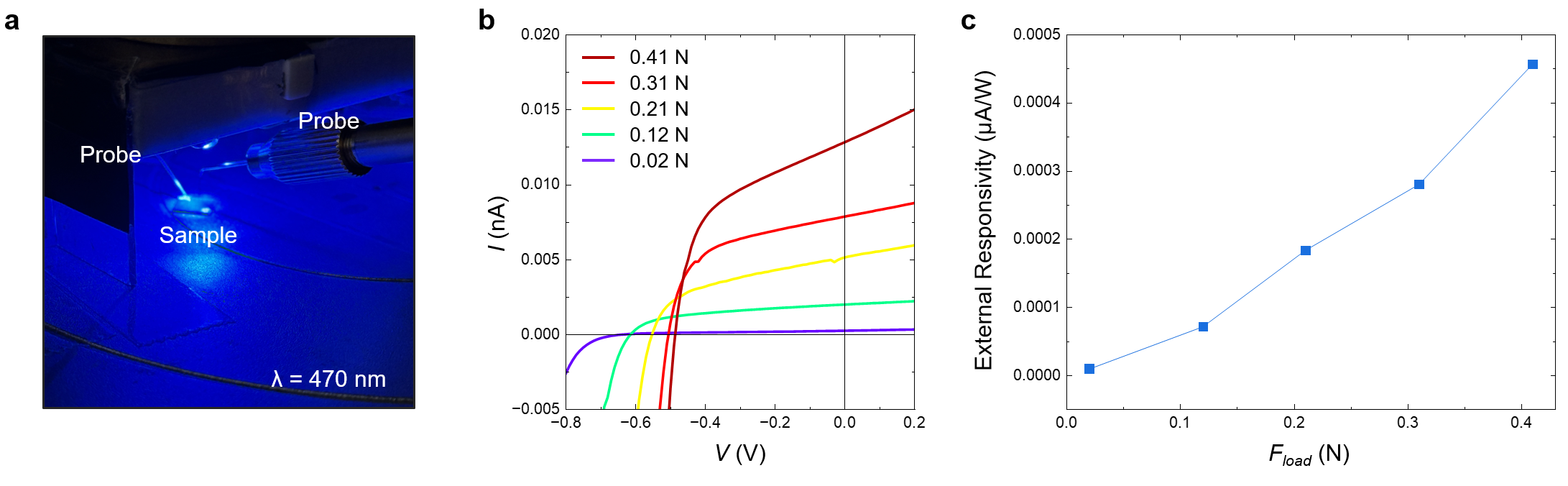


**Figure S12. *F_load_*-dependent photoresponse under blue-light illumination.** (a) Optical image of the photocurrent measurement under blue-light illumination at a wavelength *λ* of 470 nm. (b) *I-V* characteristics measured under different *F_load_*. (c) External responsivity estimated as a function of *F_load_*. Because 470 nm excitation is below the bandgap of BTO, the photoresponse can involve defect- or interface-related sub-bandgap states, and the absolute photocurrent is therefore smaller than that measured under 405 nm illumination. Nevertheless, both the photocurrent and external responsivity increase systematically with *F_load_*, confirming that the mechanically induced flexoelectric modulation remains effective in enhancing carrier extraction even when sub-bandgap photoexcitation channels are involved.

**Supplementary Note S1. Estimation of lattice strain using the Stokes-Wilson relation**

The out-of-plane lattice strain of the BTO films was estimated from the XRD *θ*-2*θ* scans using the Stokes-Wilson relation. The BTO film peak and the STO substrate peak were fitted using a Gaussian-Lorentzian peak profile to reproduce the experimental line shape. For the strain estimation, the Gaussian broadening component extracted from the fitted profile was used as the strain-related broadening term.

The corrected Gaussian broadening was calculated as

$$\beta_{G}=\sqrt{\left( \omega_{G}^{F})^{2}-(\omega_{G}^{S} \right)^{2}},$$

where $\omega_{G}^{F}$ and $\omega_{G}^{S}$ are the Gaussian widths extracted from the BTO film peak and the STO substrate peak, respectively. The strain was then estimated as

$$\varepsilon=\frac{\beta_{G}}{4\tan\theta_{B}},$$

where $\theta_{B}$ is the Bragg angle of the BTO reflection. The extracted peak widths were converted to radians before the strain calculation. The resulting strain values were used to evaluate the thickness-dependent structural relaxation of the BTO films.

**Supplementary Note S2. One-dimensional (1D) electromechanical model for flexoelectricity-enhanced BPV response**

To quantitatively examine the effect of mechanically induced flexoelectricity on the BPV response in BTO thin films, we developed a 1D electromechanical model describing the electrostatic potential distribution and carrier extraction along the out-of-plane direction (*z*). The coordinate system is defined such that the BTO surface is $z=0$, and the bottom BTO/SRO interface is $z=-t_{\mathrm{BTO}}$, where $t_{\mathrm{BTO}}$is the BTO film thickness. Therefore, the calculation domain is

$-t_{BTO}<z<0$.

We note that the mechanical stress field generated by the W-probe tip is inherently three-dimensional. The present 1D model is therefore not intended to reproduce the full spatial stress distribution around the tip contact. Instead, it provides an effective depth-dependent description of the laterally averaged out-of-plane strain gradient beneath the contact region. This approximation is appropriate for the vertical W/BTO/SRO junction because the measured photocurrent is collected along the out-of-plane direction, and the relevant flexoelectric contribution to carrier extraction is also the vertical electric-field modulation near the W/BTO interface. The validity of this effective description is further supported by the tip-radius-dependent measurements, shown in Figure 3. Both probe tips exhibit a systematic load-dependent photocurrent enhancement, while the larger-radius tip produces a weaker enhancement. This is consistent with a reduced local strain gradient rather than a simple contact-area effect. Therefore, although full three-dimensional FEM simulations could provide a more quantitative map of the local stress distribution, this 1D model captures the essential electrostatic consequence of mechanical loading: the generation of a vertical flexoelectric field that enhances interfacial carrier extraction and suppresses recombination loss.

**(1) Strain distribution and flexoelectric polarization**

Mechanical loading applied on the BTO surface induces a strain field that decays into the film. This depth-dependent strain profile reflects localized deformation beneath the W-tip contact, where the strain is largest near the top surface and gradually relaxes toward the mechanically clamped BTO/SRO interface. We assumed that the strain distribution follows

$\varepsilon\left( z \right)=\varepsilon_{0}e^{z/{l_{F}}}$, (S1)

where *l_F_* is the strain penetration length. The strain amplitude $\varepsilon_{0}$ depends on the applied mechanical loading *F_load_* and can be written as

$\varepsilon_{0}=\varepsilon_{00}\left( \frac{F_{load}}{F_{0}} \right)^{\frac{1}{3}}\left( \frac{t_{BTO}}{t_{BTO}+t_{c}} \right)$, (S2)

where *F_0_* is a reference force and *t_c_* is a thickness-coupling parameter. The resulting strain gradient is

$\frac{d\varepsilon}{dz}=\frac{\varepsilon_{0}}{l_{F}}e^{z/l_{F}}$. (S3)

Then, the flexoelectric polarization generated by this strain gradient is

$P_{F}\left( z \right)=\mu_{F}\frac{d\varepsilon}{dz}$ , (S4)

where $\mu_{F}$ is the flexoelectric coefficient. The spatial variation of polarization induces a bound charge density of

$\rho_{b}\left( z \right)=-\frac{dP_{F}}{dz}=-\frac{\mu_{F}\varepsilon_{0}}{{l_{F}}^{2}}e^{z/l_{F}}$. (S5)

**(2) Electrostatic potential and electric field**

The electrostatic potential generated by the flexoelectric bound charge is obtained from the Poisson equation including Debye screening component like

$\frac{d^{2}\varphi}{{dz}^{2}}-\frac{\varphi}{{\lambda_{D}}^{2}}=-\frac{\rho_{b}}{\epsilon_{0}\epsilon_{r}}$, (S6)

where $\varphi(z)$, $\lambda_{D}$, $\epsilon_{0}$, $\epsilon_{r}$ are the electrostatic potential, the Debye screening length, vacuum permittivity, and the relative permittivity of BTO, respectively. The short-circuit measurement condition can be imposed by the boundary conditions

$\varphi\left( -t_{BTO} \right)=0 \mathrm{and} \varphi\left( 0 \right)=0$. (S7)

We numerically solved this equation using a finite-difference scheme. The electric field distribution is obtained from the potential gradient,

$E\left( z \right)=-\frac{d\varphi}{dz}$. (S8)

**(3) Photocurrent generation**

We assume that the BPV generation profile decays exponentially with depth,

$g_{photo}\left( z \right)={g_{photo}}_{0}e^{-\frac{d}{\lambda_{abs}}}$, (S9)

where ${g_{photo}}_{0}$, $\lambda_{abs}$, and *d* are the BPV generation scale, the optical absorption length, and the distance from the surface (i.e., $d\left( z \right)=-z$), respectively. Carrier extraction is determined by the electric-field-driven drift length,

$L_{D}\left( z \right)=\left| \mu E\left( z \right)\tau\right|$, (S10)

where $\mu$ and $\tau$ are the electron mobility and the carrier lifetime, respectively. The carrier collection efficiency is defined as

$C\left( z \right)=1-e^{\left( -\frac{L_{D}}{d_{eff}} \right)}$, (S11)

where $d_{eff}\left( z \right)=d_{travel}\left( z \right)+d_{dead}$ is the effective travel distance to the electrode including a dead-layer parameter (*d_dead_*). The *d_dead_* represents interfacial recombination. Therefore, the local photocurrent contribution is

$g_{local}\left( z \right)=g_{photo}(z)C(z)$. (S12)

The photocurrent density is then obtained by integrating the local contribution across the film thickness,

$J_{photo}=\int_{-t_{BTO}}^{0} g_{local} dz$. (S13)

**(4) *F_load_*-dependence of key electromechanical and electrostatic variables**

Figure 4c to 4g show the calculated evolution of the key physical variables in the model as a function of the applied *F_load_*. For each loading condition, the strain distribution $\varepsilon(z)$ is first determined using Equation S1 and S2, from which the strain gradient and the resulting flexoelectric polarization $P_{flexo}(z)$are obtained using Equation S4. The spatial variation of the polarization generates the bound charge density $\rho_{b}(z)$ according to Equation S5. The electrostatic potential $\varphi(z)$ is then calculated by solving the Poisson-Debye equation (Equation S6) under the short-circuit boundary conditions in Equation S7. The electric field distribution $E(z)$ is obtained from Equation S8. Using the calculated electric field, the carrier drift length $L_{D}(z)$ and collection efficiency $C(z)$ are calculated according to Equation S10 and S11, and the resulting photocurrent density $J_{\mathrm{photo}}$is obtained by integrating the local photocurrent contribution across the film thickness.

Figure 4c shows the normalized photocurrent as a function of *F_load_*. Figures 4d to 4g show the calculated depth-dependent distributions of strain $\varepsilon(z)$, flexoelectric polarization $P_{flexo}(z)$, electrostatic potential $\varphi(z)$, and electric field $E(z)$, respectively.

**(5) Parameter dependence of the calculated BPV response**

To examine the robustness of the model predictions, we further evaluated the dependence of the calculated BPV response on several key model parameters while keeping the other parameters fixed. Figure 4h shows the dependence on the strain penetration length $l_{F}$, which controls the spatial extent of the mechanically induced strain field inside the BTO film. Figure 4i shows the effect of varying the Debye screening length $\lambda_{D}$, which determines the electrostatic screening of the flexoelectric bound charges. Figure 4j shows the dependence on the effective flexoelectric coefficient $\mu_{F}$, which determines the magnitude of the strain-gradient-induced polarization and the resulting interfacial electric-field modulation. Here, $\mu_{F}$ is an effective flexoelectric coefficient under our mechanical loading configuration, rather than a purely intrinsic bulk tensor component of ideal room-temperature BTO. Therefore, the examined range of $\mu_{F}$ (10^-9^ to 10^-7^ C/m) reasonably covers effective flexoelectric responses that can arise in practical ferroelectric BTO thin films under different mechanical and interfacial environments. In each case of key parameters, the photocurrent density was calculated using the same procedure described above, enabling us to evaluate how variations in the key physical parameters modify the thickness-dependent BPV response^[1-2]^.

**Supplementary References**

[1] P. Zubko, G. Catalan, A. K. Tagantsev, *Annual Review of Materials Research* **2013**, *43* (1), 387.

[2] W. Ma, L. E. Cross, *Applied Physics Letters* **2006**, *88* (23).
